# Less Economically Developed Countries Need Help to Create Healthy Workplaces

**DOI:** 10.3389/fpubh.2019.00257

**Published:** 2019-09-06

**Authors:** Midori N. Courtice, Ann C. Olsson, John W. Cherrie

**Affiliations:** ^1^Independent Post-doctoral Researcher, Edinburgh, United Kingdom; ^2^Section of Environment and Radiation, International Agency for Research on Cancer, World Health Organization, Lyon, France; ^3^Institute of Biological Chemistry, Biophysics and Bioengineering, Heriot Watt University, Edinburgh, United Kingdom; ^4^Institute of Occupational Medicine, Edinburgh, United Kingdom

**Keywords:** occupational health, occupational exposures, global burden of disease, less economically developed countries, research capacity

## Introduction

Globally, three billion working people have daily occupational health needs that can only be met by providing occupational health services, such as those to support sick workers and to ensure that workplaces are safe and healthy (1). Each year, over two million of the world's workers die from occupational diseases and 160 million get sick from non-fatal occupational diseases (2). Disability adjusted life years (DALYs) are a measure of the years of a healthy life lost from disease and premature death; in 2005 43.0 million DALYs were estimated worldwide, as a result of lung cancer, leukemia and mesothelioma (3). It is estimated that 1.6 million of those DALYs could be attributed to exposure to occupational carcinogens (4). Occupational diseases are entirely preventable, but the ILO estimates occupational injuries and diseases cost the world 2.8 trillion USD each year (5). The overall worker death rate continues to increase, with the majority of deaths taking place in poorer countries with fewer legal protections for workers (6).

Despite being disproportionately affected, it is difficult to get an accurate picture of the occupationally-related morbidity and mortality being experienced in the poorest countries of the world. One of the greatest challenges encountered while conducting occupational health research in less economically developed countries (LEDCs), is a lack of local or even regional data to describe the extent of the problem. This includes information on exposure in general and in the form of exposure measurements, and information about the industry and population under study.

## Occupational Health Publications Around the World

A search of the Scopus bibliographic database was used to identify the number of papers on occupational health across the world. Papers were searched with “occupational health” in the title, abstract or article key words. There were 76,443 documents listed; the earliest publication appeared in 1928, with around 2,500 papers published annually in recent years. A search was conducted within these to identify those where the affiliation country was within the Least Developed Countries (LDCs) as defined by the United Nations, or the Lower or Upper Middle Income Countries and Territories (LMIC and UMIC) on the Organisation for Economic Co-operation and Development's (OECD) Development Assistance Committee (DAC) list for Official Development Assistance (http://www.oecd.org/dac/financing-sustainable-development/development-finance-standards/DAC_List_ODA_Recipients2018to2020_flows_En.pdf).

In the LDCs there were between zero (34% of countries had not published a paper) and 43 papers published cumulatively between 1928 and the present; the largest number from Bangladesh, which is the most populous of this group of countries. In the LMIC and UMIC countries the range was larger: zero to 1,730 (Brazil). Unsurprisingly, the most populous countries generally published more papers related to occupational health.

**Figure 1:**
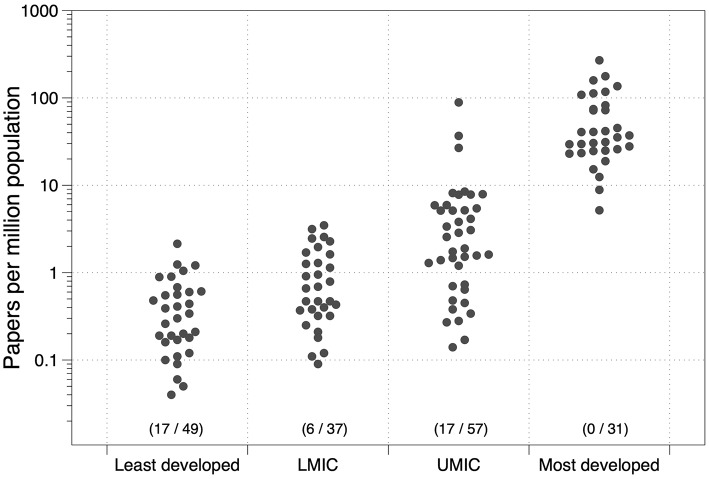
Scientific papers on occupational health from DAC countries and the most affluent nations (and the number of countries with no papers published/total countries in the group).

Figure 1 shows the data we retrieved for the DAC list countries along with comparable data for the 31 most affluent countries in the world, normalised to the total population.

Although only one bibliographic database was used to illustrate the distribution of publications between countries, we consider the distribution of publications is unlikely to be markedly different from a more comprehensive search of the literature. Clearly, the stage of development, which to a large extent is defined by the per capita gross national income, determines the ability of a country to undertake research on occupational health problems. Ninety-four of the countries in this sample of developing nations (66%) have published fewer than 10 papers on occupational health problems in the scientific literature. These data illustrate the enormous absence of knowledge about the occupational health conditions in the developing world.

## The Relative Comparability of Data and Work Situations Between Different Countries

In many cases, when conducting research studies in LEDCs, relevant data and knowledge of comparable industries from more economically developed nations are used as a proxy for local conditions and assumptions are made about the comparability of the data. Many health impact assessments take this approach as well as original research (7).

However, work situations can differ vastly between LEDCs and more industrialised nations. A larger proportion of total employment in LEDCs consists of an informal workforce comprising self-employed, household-based unpaid labour and other cottage industries or small to medium sized enterprises (fewer than 50 employees). Informal employment can be up to 70% of the total workforce compared to 10% in more industrialised nations (8). Informal workplaces are more difficult to regulate and can often have exemptions from a duty to comply with national occupational safety and health legislation. Under these circumstances more manual work practices are likely to be encountered, and with older machines where there is mechanisation, which could result in higher exposures in comparison to conditions in more industrialised nations. Most industrial exposures have decreased over time in Europe and North America with improved technologies and better control measures (9, 10), but in developing countries exposures may be increasing over time as industry expands. Workers in LEDCs may also be exposed to different hazardous agents from those described in proxy studies. They could be exposed to traditional agents such as those found in construction materials, although the silica content could differ significantly in the raw materials used in two different locations. They could also be exposed to new and emerging hazards that are not yet well-understood or characterised. Differences also exist in the availability of notification systems to record occupational diseases. Few countries have comprehensive systems for the collection of such information but they tend to be more complete in relatively more economically developed countries. In LEDCs, under-reporting of occupational diseases can occur because their registration depends on the physician recognising the link between the work and the disease, and without training in occupational medicine this may not be clearly understood. Official reporting requirements do not always cover every worker category, with the informal sector often overlooked. In some cases, the victims themselves may be worried about job security, or that there will be no support for rehabilitation or compensation (11, 12).

Assumptions about the comparability of data can lead to uncertainties and biases in the estimates of the health impacts of work exposures in LEDCs and there is a need for further intelligence, particularly in the form of exposure measurements to overcome this. If exposure is higher than that assumed from proxy data then studies of the global burden of disease may underestimate the burden from work. However, industrial development will probably have happened later in LEDCs and there would be a corresponding delay in the subsequent exposures being characterised, which could result in morbidity and mortality being overestimated. This potential for both underestimation and overestimation was described recently for the global burden of mesothelioma deaths (13). In the case of asbestos exposure, the lack of information is clear. Mesothelioma is almost exclusively attributable to past work-related asbestos exposure but mesothelioma deaths being reported worldwide do not reflect what we know about historical asbestos usage. For example, in Bangladesh and India, mesothelioma deaths have yet to be reported and yet we know there has been very high exposure to asbestos in the shipbreaking industry in these countries (14–16). Odgerel et al. estimated the global burden of mesothelioma deaths using incomplete national mortality data and discussed at length the validity of their extrapolation methods. Ultimately, global burden of disease estimates will improve as more countries improve the availability and quality of their data, and fewer countries require estimates of health impacts of work to be made from very insecure data.

## LEDCs Should be Assisted to Improve Their Occupational Health Services and Accessibility to These Services

The lack of exposure measurements from LEDCs is likely a symptom of a greater systemic problem of a lack of occupational health expertise and resources in these countries. They generally lack the resources, knowledge and technology to identify and control workplace exposure to disease-causing agents (6). Mentorship initiatives such as “Workplace Health Without Borders (WHWB)” provide valuable opportunities to engage with such local stakeholders. Established in 2011 by occupational hygienists and other activists, WHWB is an organisation that engages volunteers to improve workplace health in under-served regions of the world. Workers are provided with technical assistance, and skills and training development, to help improve and manage health conditions in their workplaces by developing the capacity and local infrastructure. They achieve this in part by working with Non-Governmental Organizations (NGOs) who serve these communities and workplaces to integrate occupational health practices into their work. Also, multifaceted mentorship approaches involving companies from relatively more economically developed countries that are operating in LEDCs could have a positive influence, and these companies should be encouraged to keep the same high standards of working conditions as in their country of origin.

LEDCs must be assisted to improve their occupational health services and accessibility to these services. We believe there is an obligation for more economically developed nations to assist LEDCs in accordance with the UN Sustainable Development Goals (SDGs). These consist of 17 broad-based goals addressing issues related to poverty, inequality, climate, environmental degradation, prosperity, and peace and justice. Several of the goals support the right to healthy workplaces:

Goal #3 Good Health and Well-Being:

By 2030, substantially reduce the number of deaths and illnesses from hazardous chemicals and air, water, and soil pollution and contamination.
Strengthen the capacity of all countries, in particular developing countries, for early warning, risk reduction and management of national and global health risks.

Goal #8 Decent Work and Economic Growth:

Protect labour rights and promote safe and secure working environments of all workers, including migrant workers, particularly women migrants, and those in precarious employment.

Goal #16 Peace, Justice and Strong Institutions:

Develop effective, accountable and transparent institutions at all levels.

Goal #17 Partnerships for the Goals.

Strengthen the means of implementation and revitalize the global partnership for sustainable development.

## Conclusion

When attempting to quantify the burden of death and disease being experienced globally as a result of workplace exposures, as we have discussed, it is commonplace to use research from more industrialised nations to represent conditions in LEDCs. These efforts should not be dismissed as much thought and effort can go into accounting for differences between the two study locations and any assumptions made will have been clearly defined. However, the absence of knowledge about workplace exposures in LEDCs is vast and must lead to imprecise and perhaps underestimation of occupational disease burden. LEDCs should be encouraged to increase their capacity for occupational health research. More economically developed countries have a responsibility to assist LEDCs to improve their occupational health services in accordance with the UN SDGs, to provide healthy and safe workplaces for all, by 2030. We recommend supporting mentorship initiatives that use a grassroots approach to connect experienced occupational health professionals with key local stakeholders in LEDCs. We consider that developed countries should prioritise some of their official development aid to help improve capability to assess and control workplace exposures, improve occupational health, and support research.

## Author's Note

Where authors are identified as personnel of the International Agency for Research on Cancer/World Health Organization, the authors alone are responsible for the views expressed in this article and they do not necessarily represent the decisions, policy or views of the International Agency for Research on Cancer/World Health Organization.

## Author Contributions

MC, JC, and AO contributed to the text throughout the manuscript. JC provided the figure.

### Conflict of Interest Statement

The authors declare that the research was conducted in the absence of any commercial or financial relationships that could be construed as a potential conflict of interest.
